# Supplementary material for: Chromothripsis during telomere crisis is independent of NHEJ, and consistent with a replicative origin
Source: Genome Res. 2019 May;29(5):737–49. doi: 10.1101/gr.240705.118 (PMC6499312; doi:10.1101/gr.240705.118)
Supplement: Supplemental Material [file supp_gr.240705.118_Supplemental_file_1.zip › contigs/annotated_contigs/DB79/contig.2.DB79_length_450_mean_cov_16.8733333333.docx]

**DB79_length_450_mean_cov_16.8733333333**

```
GGCAGGTG|GATCACCTGAGGTCAGGAGTTCGAGACCAGCCTGGCCAACATGGTGAAACCCCATCTCTACTANAAATACAAAATTAGTG
         >chr14:42203299-42203456 - E=8e-41 p=0e+00
CTCACCTGTGATCCCAGCTACTCAGGAGGCTGAGGCATGAGAATCACTTGAACCTAGGAGGCAGAGTTTGCTGTGAG|TTTGCTAAGAG

TTTG|CTAAGAACTGAACATATCCTGTCCTCCTAAAATTGGTTTGTGTTTGAGGGAAATTATCTAAAGACTTGAGTAGTGGCTGCATAT
     >chr20:35935519-35935789 - E=5e-151
TACAATCACCTGGACACTTTAAAAAAATACTGATCCAAGCCGGGCACACTGGCTCATGCCTGTAATCCCATCACTTTGGGAGGCCAAGG

TGAGAGAATCACCTGAGCCCAGGAGTTCAAGACCAGCCTGGGCAACATAGTGAAACCGTGTCTCTACAAAAAAATTATTTAAAAATTAG

CCAGGTGT
```
